# Severe Cutaneous Reaction Induced by Clindamycin: A Case Report of Toxic Epidermal Necrolysis

**DOI:** 10.7759/cureus.70098

**Published:** 2024-09-24

**Authors:** Gabriel Scally, Yohannes Haile, Allen Seylani, Nicholas W Sheets

**Affiliations:** 1 General Surgery, Riverside Community Hospital, Riverside, USA; 2 Surgery, University of California Riverside School of Medicine, Riverside, USA; 3 Trauma and Acute Care Surgery, Riverside Community Hospital, Riverside, USA

**Keywords:** clindamycin, drug-induced hypersensitivity, medical dermatology, severe cutaneous adverse reaction, toxic epidermal necrolysis (ten)

## Abstract

Toxic epidermal necrolysis (TEN) is a rare, acute inflammatory skin reaction that results in skin blistering and extensive epidermal detachment. Stevens-Johnson syndrome (SJS) and TEN are unified aspects on a spectrum varying in the severity of vesiculobullous cutaneous eruptions with mucosal involvement of the oral cavity, genitourinary tract, gastrointestinal tract, and conjunctiva. The inciting event is usually caused by an exaggerated hypersensitivity reaction in response to triggering medications, including nonsteroidal anti-inflammatory drugs (NSAIDs), antibiotics, urate-lowering drugs (such as allopurinol), anticonvulsants, and antipsychotics. We report a case of clindamycin-induced TEN in a 79-year-old African-American female following the recent administration of clindamycin for a developing sacral decubitus ulcer. However, lincosamide antibiotics like clindamycin are rarely associated with precipitating SJS or TEN. This report highlights the treatment and prognostic challenges faced throughout the patient’s clinical course and seeks to highlight the importance of recognizing the development of SJS/TEN following novel drug administration and promptly addressing the management of the condition to improve long-term patient outcomes.

## Introduction

Stevens-Johnson syndrome (SJS) and toxic epidermal necrolysis (TEN) are rare autoimmune reactions that account for approximately one to two cases per 1,000,000 people per year, which offending agents, such as medications or infections, can trigger [1]. These disease processes are characterized by vesiculobullous lesions that can be widespread, including skin sloughing, and are not just limited to the skin. The genitourinary tract, gastrointestinal tract, oral cavity, and conjunctiva can also be involved. Occasionally, a prodromal phase of fever, malaise, and upper respiratory tract symptoms can be seen [2]. SJS and TEN remain a clinical diagnosis, yet confirmation with a biopsy may reveal full-thickness epidermal necrosis [3]. The extent of skin involvement determines the categorization of these diseases, where SJS involves <10% of total body surface area (TBSA) and TEN involves >30% of TBSA [1]. Most cases are primarily attributed to certain medications, including phenobarbital, phenytoin, carbamazepine, lamotrigine, nonsteroidal anti-inflammatory drugs (NSAIDs), allopurinol, and fluconazole [2]. *Mycoplasma pneumoniae* and herpes simplex virus have even been implicated [1]. TEN and SJS can be associated with particular genetic susceptibilities, most notably between the HLA-B*1502 allele and carbamazepine use among the Han Chinese and Thai populations [1]. It is crucial to promptly identify early signs and symptoms from other differential diagnoses such as erythema multiforme, staphylococcal scalded skin syndrome (SSSS), drug rash with eosinophilia and systemic symptoms (DRESS), and acute graft versus host disease [1]. While immediate cessation of the offending agent is crucial to prevent disease progression, the treatment for TEN and SJS is mainly supportive, with limited evidence regarding medical management [1]. The prognosis of SJS and TEN is variable, and mortality risk can range from 1-5% and 25-35%, respectively [1].

Clindamycin, an antibiotic in the lincosamide family that inhibits bacterial protein synthesis, has been used in treating multiple conditions, including lower respiratory infections, gynecological infections, and skin infections [4]. While uncommon, adverse reactions associated with clindamycin include pruritus, erythema, and burning of the skin [4]. One of the more severe complications of clindamycin use is the development of pseudomembranous colitis secondary to *Clostridioides difficile* infection [4]. TEN development after clindamycin therapy is scarce, and few reports have illustrated a potential cause, yet this case describes the rare instance of their connection.

## Case presentation

**Figure 1:**
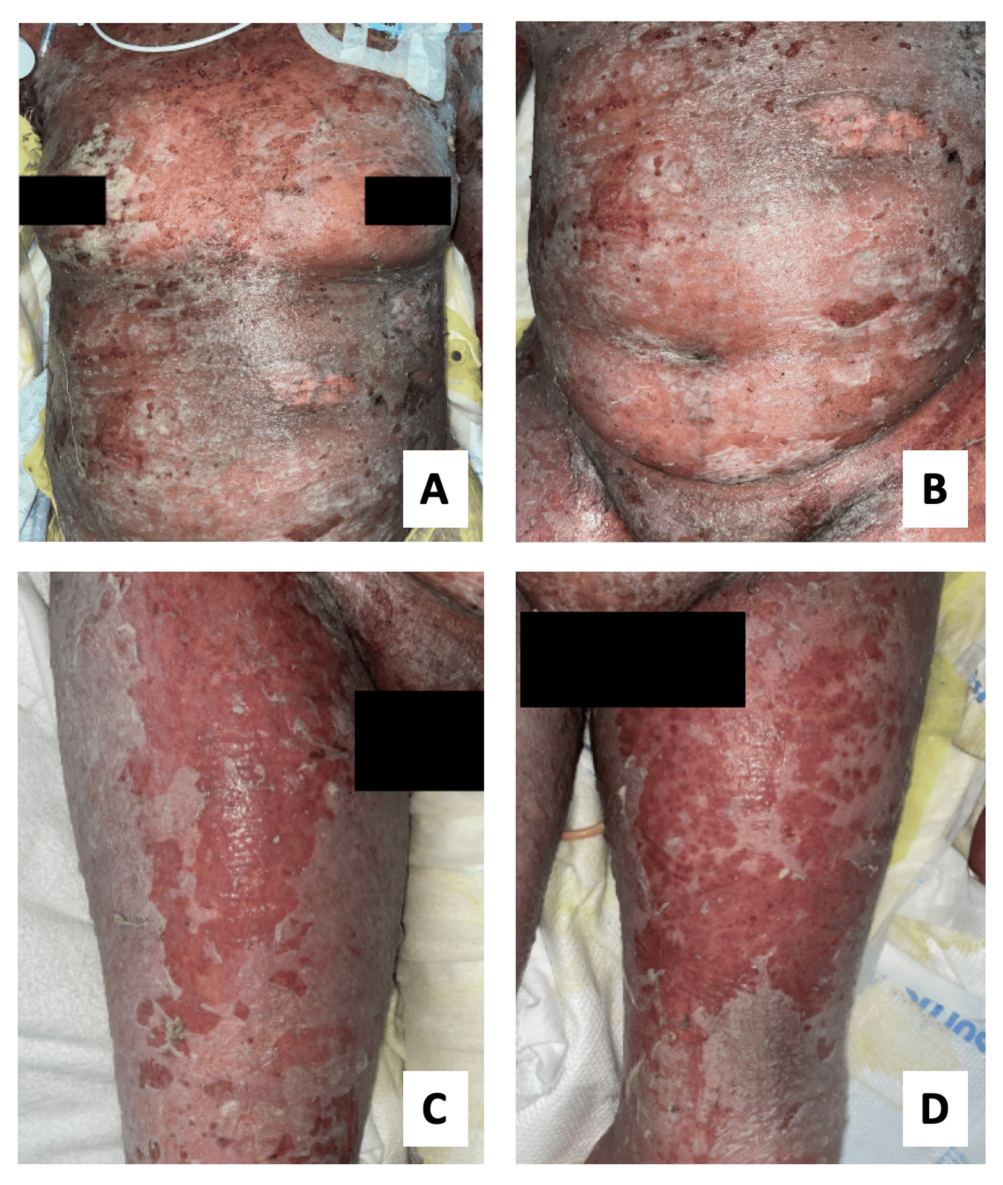
Coalescent erythematous macular rash with sheets of denuded epidermis seen on the chest (A), abdomen (B), and bilateral lower extremities (C, D).

A 79-year-old African-American female with a medical history encompassing type 2 diabetes, congestive heart failure (CHF), and chronic kidney disease (CKD) presented to her primary care provider with a persistent pressure ulcer on her left buttock. After collecting a wound culture, clindamycin was administered as the chosen antibiotic. Initially, there was a noticeable improvement in the infection; however, days after initiating antibiotic therapy, the patient developed a widespread maculopapular rash, prompting immediate cessation of clindamycin and presentation to the emergency department (ED). On presentation, the patient was noted to be septic with a diffuse maculopapular rash involving the abdomen and extremities with altered mental status. The patient’s vitals were significant for a heart rate of 93 and blood pressure of 94/54 with significant laboratory results of white blood cell count of 28, blood urea nitrogen (BUN) of 68, creatinine of 3.33, bicarbonate of 20, glucose of 265, and lactic acid of 4.1. The patient was subsequently admitted for treatment with broad-spectrum antibiotics and fluid resuscitation. On hospital day 2, a skin biopsy revealed subepidermal vesicle formation with necrosis, lymphocytic invasion, and perivascular inflammation consistent with SJS and TEN. Notably, the maculopapular rash migrated to the torso and head with developing mucosal involvement. The original rash rapidly progressed within hours to skin sloughing with exposed raw dermal surfaces (Figure 1).

The patient was subsequently upgraded to the intensive care unit (ICU) due to episodes of hypotension intermittently responsive to crystalloid boluses. On hospital day 3, the patient’s neurological status declined, requiring intubation. During her stay in the ICU, the patient developed worsening renal function in addition to her baseline CKD, subsequently requiring hemodialysis. Throughout her admission, the patient’s skin condition deteriorated further, eventually encompassing 50% TBSA (Figure 1). The patient’s wounds were covered with petroleum-impregnated gauze and topical 1% hydrocortisone cream twice daily, with minimal overall improvement. The patient received IV steroids early in her ICU stay, again with limited improvement. Episodes of melena and vaginal bleeding also occurred, which likely represented the extensive mucosal involvement of the disease process. During the patient’s stay in the ICU, her clinical status slowly deteriorated with multiple episodes of cardiac arrest secondary to cardiac etiology due to her long-standing history of CHF, with a return of spontaneous circulation (ROSC). The patient had a prolonged hospital course and developed multiorgan failure with no signs of improvement. Ultimately, the family decided to transition the patient to comfort care, and she expired shortly thereafter.

## Discussion

TEN is a lethal disease typically triggered by sulphonamides, barbiturates, anticonvulsants, urate-lowering drugs, NSAIDs, and antibiotics [2]. Penicillins and their derivatives are commonly implicated as the most common causes of TEN among all antibiotics [5]. Nevertheless, clindamycin-induced TEN is highly uncommon, with only four previous cases documenting this correlation [6,7]. Previously reported cases of clindamycin-induced TEN occurred in healthy individuals who ultimately achieved resolution of symptoms within three to four weeks without significant long-term morbidity with no specific intervention [6,7]. However, our patient presented with multiple comorbidities, which significantly increased her mortality risk upon admission. This is the first case describing clindamycin-induced TEN leading to death, which is novel.

TEN starts with a prodromal phase of fever and cough followed by subsequent eruption of morbilliform, erythematous, and atypical macules [1]. Furthermore, TEN patients develop flaccid bullae, skin erosions, and ulcerated mucosal lesions one day to two weeks after initiating the offending agent [1]. Mucosal involvement is diffuse and can involve multiple organ systems such as the oral cavity, gastrointestinal tract, and genitourinary system [1]. Gastrointestinal bleeding and colonic perforation are severe complications that rarely occur. Acute genital manifestations, including vulvar bullae and erosions, vaginal bleeding, or secondary infection, have also been documented [8]. The development of melena in our patient likely points to TEN-associated mucositis of the gastrointestinal tract. These findings highlight the challenges in managing TEN-associated complications due to multiorgan involvement exacerbated by systemic inflammation.

A thorough evaluation, including history and physical exam, skin biopsy, laboratory studies, and early specialty consultation for a multidisciplinary and comprehensive assessment, is vital in obtaining and assessing the diagnosis of TEN. These aspects allowed our team to differentiate our patient’s symptoms from other differentials, including but not limited to SSSS and DRESS. Body surface area (BSA) was assessed in our patient using the Lund-Browder chart to monitor signs of improvement or decline. The study by Bastuji-Garin et al. proposes using an illustrated atlas after conducting physical exams to standardize the diagnosis of TEN and other bullous skin disorders, which could have been utilized in our patient to facilitate better multidisciplinary care [9]. BSA has been used as a surrogate for prognosis in patients with TEN, meaning as BSA increases, so does mortality.

The severity-of-illness score for toxic epidermal necrolysis (SCORTEN) and ABCD-10 (age, bicarbonate, cancer, dialysis, and 10% BSA) prognostic tools utilize physical exam findings and laboratory studies as variables to predict patient outcome. The SCORTEN system follows seven independent risk factors for death, including age >40, malignancy, tachycardia above 120/min, initial percentage of epidermal detachment >10%, serum urea above 28 mg/dL, serum glucose above 252 mg/dL, and bicarbonate below <20/liter [10]. SCORTEN scores have higher discriminatory power and agreement between expected and actual mortality compared to other scoring systems [10]. SCORTEN scores 0 to 1 indicate a mortality risk of ~3%, with >5 indicating a 90% mortality risk.

**Figure 2:**
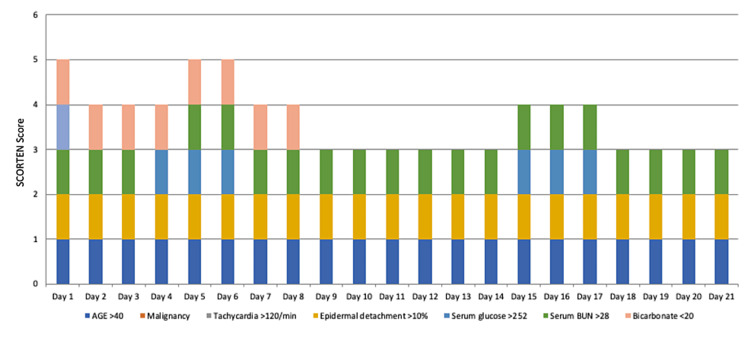
The patient's SCORTEN score throughout the entire admission SCORTEN values and reported mortality risk percentages are as follows: 0-1 = 3%, 2 = 12%, 3 = 35%, 4 = 58%, 5 or more > 90%. SCORTEN: severity-of-illness score for toxic epidermal necrolysis; BUN: blood urea nitrogen

Our patient’s SCORTEN score was tabulated for each day of admission and displayed in Figure 2. The significant escalation observed on day 1 and days 5 and 6, with scores peaking at 5, cannot be underestimated as it was an important indicator of our patient’s prognosis. The study by Guégan et al. revealed significant differences between days 1 and 4, with more accurate estimation on day 3 when comparing the number of deaths expected using the SCORTEN system and the outcome observed at discharge [11]. Furthermore, a strong correlation was identified (with validity exceeding 80%) between the anticipated and actual number of deaths during the first five days of admission, emphasizing the importance of considering this entire period instead of relying solely on scores from day 1 [11]. This observation may be attributed to the spectrum of disease severity among individual patients upon admission. Our support for this notion is based on our patient’s clinical presentation and complexity upon admission and throughout her hospital stay.

The association between CKD and TEN is a topic of emerging interest in dermatology and nephrology. Recent studies have suggested a potential link between CKD and the development and exacerbation of TEN, although the underlying mechanisms remain unclear [12]. It is hypothesized that impaired renal clearance in CKD patients may lead to the accumulation of the offending medications and their metabolites, thereby increasing the risk of TEN [12]. Understanding this association is crucial for optimizing patient care, particularly in the management of medication therapies and the prevention of adverse drug reactions in this vulnerable population. While CKD itself is associated with increased mortality due to its systemic effects and complications, some studies have suggested that CKD may be a comorbidity that exacerbates the severity of TEN and complicates its management, potentially leading to higher mortality rates [13]. Additionally, CKD may alter the pharmacokinetics of medications used in TEN treatment, which could influence patient outcomes [12]. However, more research is needed to establish a direct causal relationship between CKD and increased mortality in TEN patients and to elucidate the underlying mechanisms.

**Figure 3:**
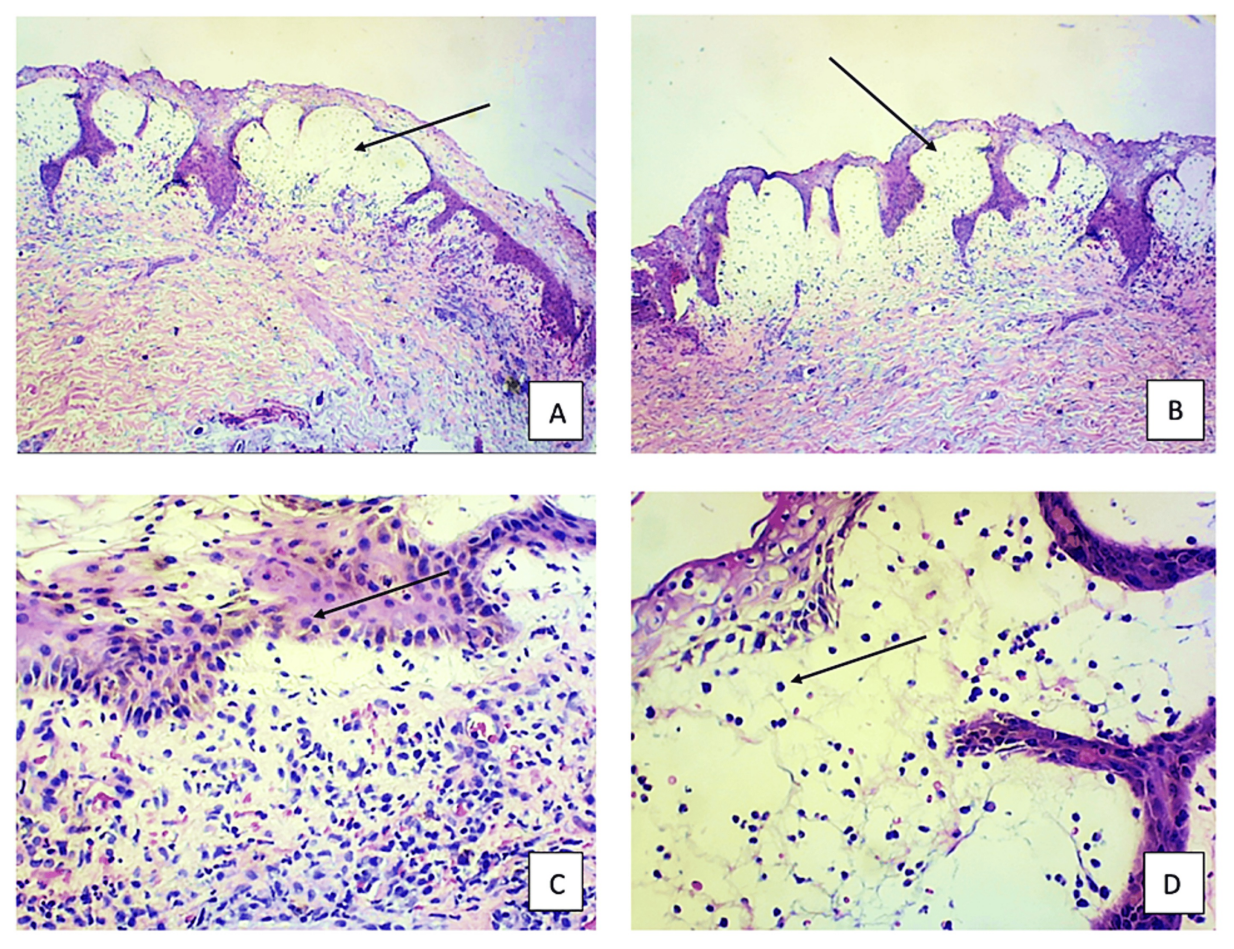
Histology slides from a skin biopsy obtained from the patient's right upper extremity A 5x low power field histology showing subepidermal vesicle formation indicated by arrows (A & B). A 10x high power field histology showing predominance of lymphocytes indicated by arrows (C & D).

The histological examination of TEN reveals full-thickness necrosis of the epidermis, separation of the epidermis at the dermo-epidermal junction, dermal inflammatory cell infiltrations, and peri-appendageal inflammatory cell infiltrates (Figure 3) [3]. Keratinocyte apoptosis forms characteristic skin blisters and denudations that involve the skin and mucous membranes. As BSA increases, mortality also increases, but the degree of full-thickness epidermal necrosis does not independently have enough predictive power to elucidate mortality [3]. As the histopathological severity of epidermal necrosis has not been able to predict survival in TEN patients, future studies should investigate a combined clinical and histologic scoring system to calculate mortality risk.

SJS and TEN have the same underlying pathophysiology, only differing in BSA. Multiple pathophysiological mechanisms have been proposed, and several biomarkers that could be used as diagnostic tools have been identified. The cause of TEN is a consequence of a delayed T-cell-mediated reaction (type-4 hypersensitivity) [2]. The study by Abe et al. proposes that peripheral mononuclear cells are responsible for the release of Fas ligand (FasL) activated by the offending antigen, in our case, clindamycin, which binds to Fas on keratinocytes and causes apoptosis induced by cytotoxic T lymphocytes (CTL) and natural killer (NK) cells [14]. Granulysin, an essential protein in apoptosis, is released from CTL and NK cells and has been interpreted as the defining mediator in keratinocyte apoptosis [15]. The study by Chung et al. reported that granulysin exhibited significantly higher concentrations than FasL levels found in the fluid from the skin blisters biopsied [15]. In future studies, it is crucial to investigate the role of lymphocytes in delineating the most accurate mechanism for TEN to determine diagnostic and therapeutic treatment approaches.

As discussed previously, the prognosis of TEN patients can be predicted using the SCORTEN scoring system. Clinical and histological diagnosis of SJS/TEN should guide initial management as TBSA >35%, age >40, and the presence of comorbid illness requires immediate transfer to a burn center [16]. However, TBSA <10% can be treated in a non-burn center with monitoring of possible disease progression. Treatment includes systemic corticosteroids, IVIg, cyclosporine, plasmapheresis, anti-tumor necrosis factor (TNF) alpha drugs, and supportive wound care [17]. Though improvements in mortality have been noted with some therapies, none have been established as the standard of care overall. In the absence of definitive treatment, Han et al. sought to find a consensus for treatment using a survey among dermatologists across the United States, yet their findings revealed significant heterogeneity [18]. This highlights the importance of future studies to investigate both the biological and clinical aspects of adjunct treatments for TEN.

Wound care management consists of daily dressing changes with possible debridement and biologic dressings such as allografts, xenografts, or biosynthetic dressings. Biosynthetic skin substitutes have shown improved outcomes over conservative antiseptic wound treatment with insignificant changes in mortality rates [19]. Nevertheless, early wound coverage with such skin substitutes has shown reduced pain, improved mobilization, and decreased risk of infection [19]. These approaches seek to promote skin regeneration for acute disease abatement and prevent future complications of TEN, as over half of survivors experience long-term sequelae of the disease ranging from changes in pigmentation to scar formation [1].

Clindamycin-induced TEN is an infrequent finding, making it essential to understand how to improve care for this small patient population. Obtaining a diagnosis early must be emphasized because when epidermal necrolysis is suspected, swift action in management improves patient outcomes. Furthermore, this case seeks to address the underrepresentation of patients with darker skin tones and reports of TEN in medical literature and educational resources. This becomes apparent when considering patients with higher Fitzpatrick phototype scores, a classification system of the skin by its reaction to sunlight exposure, have a 4% increased risk of mortality. These findings may be due to differences in biological attributes necessitating specific therapeutic measures or a delay in diagnosis resulting from limited resources for dermatologic diseases in different skin colors in the literature [20]. Lastly, the capacity to utilize a specific biomarker for precise diagnosis of TEN would mark a crucial advancement in caring for this patient population, distinguishing TEN from less severe conditions. While we employed the SCORTEN tool to assess the prognosis and comprehend our patient's condition, its use in cases with multiple comorbidities may inaccurately skew patients’ prognosis. Therefore, a more comprehensive scoring system could better gauge the sensitivity of a patient’s clinical status.

## Conclusions

This case of clindamycin-induced TEN in an elderly patient with multiple comorbidities highlights the challenges in managing this life-threatening condition. Despite aggressive management efforts, including early recognition, cessation of the offending agent, and supportive care, the patient's clinical course rapidly deteriorated, ultimately leading to multiorgan failure and death. This provides evidence of how quickly this disease process can lead to significant morbidity and mortality. The rarity of clindamycin-induced TEN, coupled with the patient's advanced age and existing medical conditions, underscored the case's complexity.

Our case emphasizes the importance of early recognition and prompt intervention in suspected cases of TEN and the need for greater awareness and education. The association between CKD and TEN warrants further investigation to better understand its implications for patient care and outcomes. Moving forward, continued research into the pathophysiology, diagnosis, and management of TEN is essential to improve outcomes and reduce mortality rates. Additionally, efforts to enhance education and awareness of TEN among healthcare providers, particularly regarding its presentation in elderly patients and those with multiple comorbidities, are crucial for timely diagnosis and appropriate management. Furthermore, addressing the underrepresentation of individuals with darker skin tones in medical literature and resources is essential for ensuring equitable access to healthcare and improving outcomes for all patients affected by TEN. Overall, this case serves as a poignant reminder of the devastating impact of TEN and the urgent need for further research and improved therapeutic strategies to address this life-threatening condition effectively.

## References

[1] Harr T, French LE (2010). Toxic epidermal necrolysis and Stevens-Johnson syndrome. Orphanet J Rare Dis.

[2] Frantz R, Huang S, Are A, Motaparthi K (2021). Stevens-Johnson syndrome and toxic epidermal necrolysis: a review of diagnosis and management. Medicina (Kaunas).

[3] Chuenwipasakul D, Washrawirul C, Panpruk R (2023). Correlations between histopathologic findings, serum biomarker levels, and clinical outcomes in Stevens-Johnson syndrome/toxic epidermal necrolysis (SJS/TEN). Sci Rep.

[4] Murphy PB, Le JK (2024). Clindamycin. StatPearls.

[5] Frey N, Bircher A, Bodmer M, Jick SS, Meier CR, Spoendlin J (2018). Antibiotic drug use and the risk of Stevens-Johnson syndrome and toxic epidermal necrolysis: a population-based case-control study. J Invest Dermatol.

[6] Davey MG, Birrane J, Brennan M, Breen DP, Laing ME (2020). Clindamycin induced toxic epidermal necrolysis versus Staphylococcal scalded skin syndrome: a case report. Oxf Med Case Reports.

[7] Paquet P, Schaaf-Lafontaine N, Piérard GE (1995). Toxic epidermal necrolysis following clindamycin treatment. Br J Dermatol.

[8] Gulanikar A, Abrol A, Sagar S (2022). Study of genital manifestations of Stevens Johnson syndrome/toxic epidermal necrolysis. Indian J Sex Transm Dis AIDS.

[9] Bastuji-Garin S, Rzany B, Stern RS, Shear NH, Naldi L, Roujeau JC (1993). Clinical classification of cases of toxic epidermal necrolysis, Stevens-Johnson syndrome, and erythema multiforme. Arch Dermatol.

[10] Koh HK, Fook-Chong S, Lee HY (2020). Assessment and comparison of performance of ABCD-10 and SCORTEN in prognostication of epidermal necrolysis. JAMA Dermatol.

[11] Guégan S, Bastuji-Garin S, Poszepczynska-Guigné E, Roujeau JC, Revuz J (2006). Performance of the SCORTEN during the first five days of hospitalization to predict the prognosis of epidermal necrolysis. J Invest Dermatol.

[12] Mathur M, Thakur N, Jaiswal S, Maharjan S, Paudel S, Shrestha A (2023). Recurrent toxic epidermal necrolysis with two different drugs in a patient with chronic kidney disease-a case report with an atypical presentation. Clin Case Rep.

[13] Rattanakaemakorn P, Palakornkitti P, Pinyowiwat P, Jedee P, Thadanipon K (2022). Chronic kidney disease is potentially an independent prognostic factor for death in Stevens-Johnson syndrome and toxic epidermal necrolysis patients. Front Med (Lausanne).

[14] Abe R, Shimizu T, Shibaki A, Nakamura H, Watanabe H, Shimizu H (2003). Toxic epidermal necrolysis and Stevens-Johnson syndrome are induced by soluble Fas ligand. Am J Pathol.

[15] Chung WH, Hung SI, Yang JY (2008). Granulysin is a key mediator for disseminated keratinocyte death in Stevens-Johnson syndrome and toxic epidermal necrolysis. Nat Med.

[16] Ellis MW, Oster CN, Turiansky GW, Blanchard JR (2002). A case report and a proposed algorithm for the transfer of patients with Stevens-Johnson syndrome and toxic epidermal necrolysis to a burn center. Mil Med.

[17] Schmidt V, Lalevée S, Traidl S (2023). Intravenous immunoglobulins, cyclosporine, and best supportive care in epidermal necrolysis: diverse effects on systemic inflammation. Allergy.

[18] Han JJ, Creadore A, Seminario-Vidal L, Micheletti R, Noe MH, Mostaghimi A (2022). Medical management of Stevens-Johnson syndrome/toxic epidermal necrolysis among North American dermatologists. J Am Acad Dermatol.

[19] Boorboor P, Vogt PM, Bechara FG, Alkandari Q, Aust M, Gohritz A, Spies M (2008). Toxic epidermal necrolysis: use of Biobrane or skin coverage reduces pain, improves mobilisation and decreases infection in elderly patients. Burns.

[20] Wasuwanich P, So JM, Chakrala TS, Chen J, Motaparthi K (2023). Epidemiology of Stevens-Johnson syndrome and toxic epidermal necrolysis in the United States and factors predictive of outcome. JAAD Int.

